# Supplementary material for: Structural Bias in Three-Dimensional Autoregressive Generative Machine Learning of Organic Molecules
Source: J Chem Inf Model. 2025 Jun 25;65(13):6644–54. doi: 10.1021/acs.jcim.5c00665 (PMC12264931; doi:10.1021/acs.jcim.5c00665)
Supplement: Supplementary file 1 [file ci5c00665_si_001.pdf]

Structural bias in three-dimensional autoregressive generative machine learning of organic molecules

Zsuzsanna Koczor-Benda,[†] Francesco Bartucca,[†] Abdulla Al-Fekaiki,[†] Joe Gilkes,[†,‡] and Reinhard J. Maurer[*,†,¶]

†*Department of Chemistry, University of Warwick, Coventry, CV4 7AL, United Kingdom*

‡*Centre for Doctoral Training in Modelling of Heterogeneous Systems, University of Warwick, Coventry, CV4 7AL, United Kingdom*

¶*Department of Physics, University of Warwick, Coventry, CV4 7AL, United Kingdom*

E-mail: r.maurer@warwick.ac.uk

G-SchNet hyperparameter settings

We have used G-SchNet version 1.0.0, commit: ecc48bcea85c88a415c6a98ee7f62db73ecc6031 (Nov 7, 2023). Tables S1 and S2 list some of the settings used for training and generation for the QM9 and OE62 datasets. All other settings were kept at the default values.

Table S1. Training parameters used for models trained on QM9 and OE62 datasets. Units are the default units as expected by G-SchNet, i.e. Å for distances.

	QM9	OE62
model_cutoff	10.0	10.0
prediction_cutoff	10.0	10.0
placement_cutoff	1.7	2.6
use_covalent_radii	true	true
covalent_radius_factor	1.1	1.3
lr	0.0001	0.0001
draw_random_samples	0 (generate all paths)	5
batch_size	5	5
num_train	50,000	45,000
num_val	5,000	4,500

Table S2. Generation parameters used for models trained on QM9 and OE62 datasets. Units are the default units as expected by G-SchNet, i.e. Å for distances.

	QM9	OE62
n_molecules	60,000	60,000
batch_size	1	1
max_n_atoms	35	200
grid_distance_min	0.7	0.7
grid_spacing	0.05	0.05
temperature_term	0.1	0.1
grid_batch_size	0	0

Downsampling of generated datasets

Where specified in the main text, datasets of generated molecules were downsampled such that their distributions of a target property (in this case the number of atoms in each molecule) became aligned with the distribution of this property in the training dataset. This uses a custom algorithm which is implemented in our GSchNetTools Python package, linked in the main text.

Downsampling is achieved by first binning the training dataset's target property into a histogram — in all cases within this work, we used a bin width of 10 for atom count distributions. Each bin's probability density is calculated based on the number of molecules that falls within it. Generated datasets are similarly binned, and each bin is then sampled without replacement to yield a probability density as similar as possible to its respective bin density from the training set. We use the probability density of each bin as the target because it is normalised, thus allowing for generated datasets of different sizes to the training set to be sampled.

Additional data for model trained on QM9 training data

Generated Molecule Analysis

Table S3. Number of unique and valid molecules generated with different G-SchNet settings, based on the QM9 training dataset.

Trajectories	Train size	Target	Generated	Duplicate	Disconnected	Filtered
All	10000	60000	59832	0	8587	39256
All	30000	60000	58479	0	8609	38194
All	50000	60000	59165	0	10271	37624
5	10000	60000	59630	0	10575	38163
5	30000	60000	59866	0	11537	38531
5	50000	60000	59882	0	10616	38190

The QM9 training database contains 130831 molecules, out of which 128765 can be converted into valid RDKit molecules. The G-SchNet model trained on all atom placement trajectories and with a training data set size of 50000 generates 35551 molecules for which RDKit is able to generate valid SMILES strings.

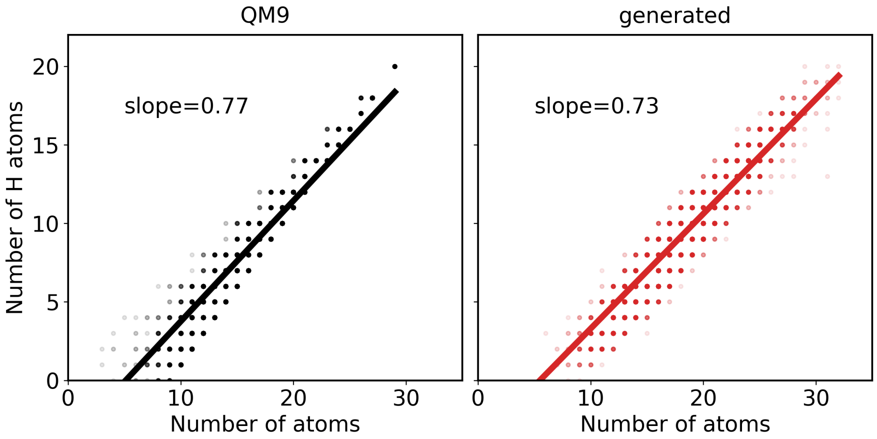

Figure S1. Number of H atoms as a function of number of atoms for the QM9 straining data (left) and the G-SchNet generated molecules (right). The dots indicate the distribution and the line corresponds to a trend line fitted to this distribution.

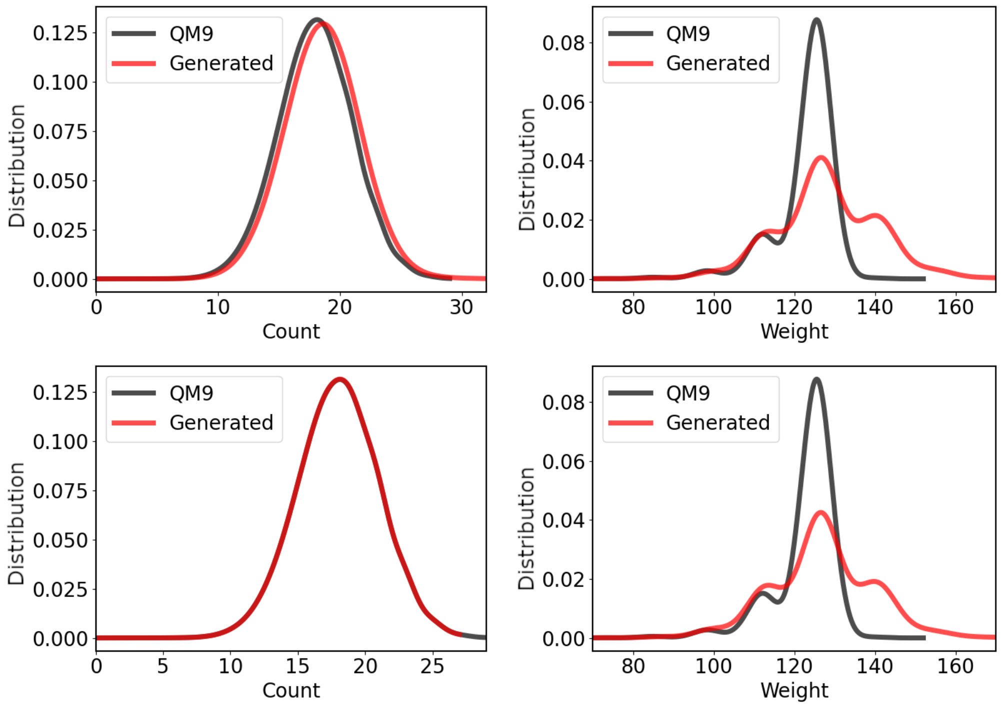

Figure S2. Distribution of total atom counts and molecular weights in QM9 training and generated molecules. Top panels: comparison of QM9 training data and original generated molecule database, Bottom panels: comparison of QM9 training data and the generated dataset after resampling according to the training set's atom count distribution.

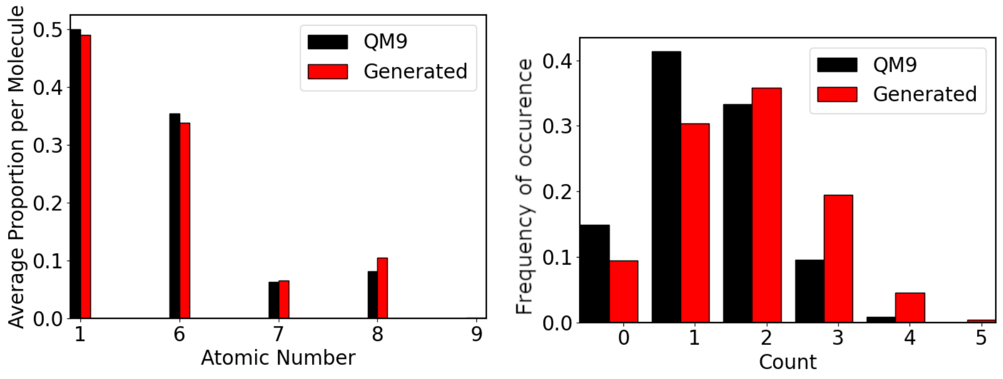

Figure S3. Left: Elemental composition of QM9 and generated molecular databases shown as average proportion of elements per molecule. Right: Frequency of occurrence of oxygen atoms in QM9 and generated molecules.

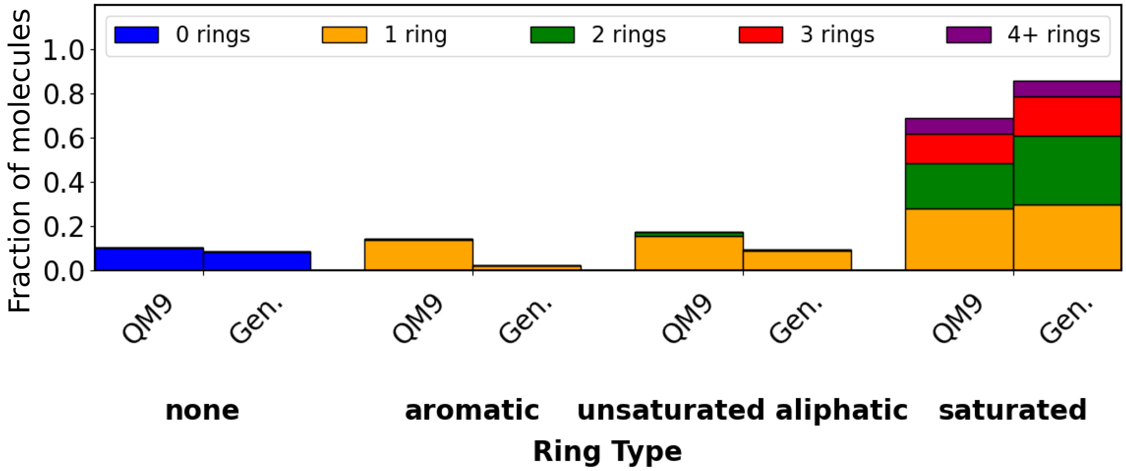

Figure S4. Proportion of QM9 training and generated molecules containing rings, separated into different ring types and counts.

Decision Trees

For training decision tree discriminators, we generated Morgan fingerprints with radius 0 and number of bits 1000. When also including atom counts in the descriptor, the optimal tree depth is 15 with validation score 0.740 (Figure S5). Without atom counts, the optimal tree depth is 11 with validation score 0.681. Thus including atom counts in the feature vector results in significantly more accurate predictions, and the former model is used in the analysis below.

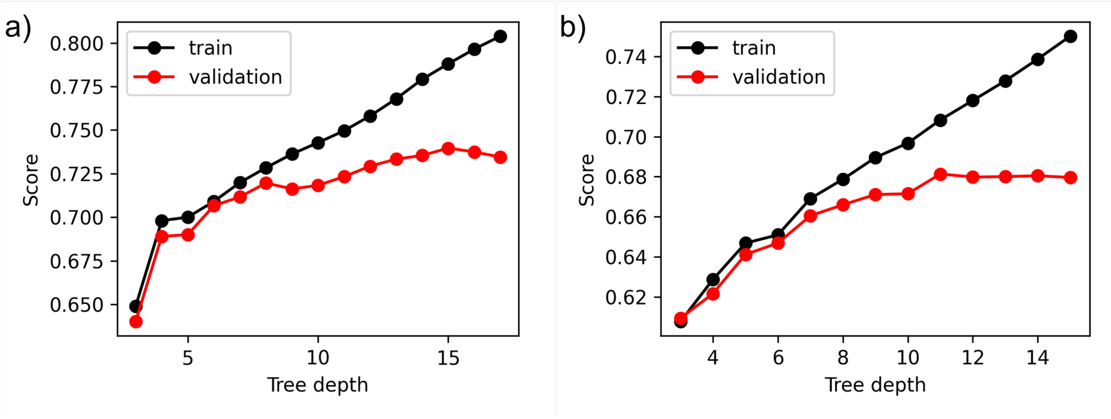

Figure S5. Optimising the tree depth parameter of a decision tree model to discriminate between QM9 and generated molecules. Features include a) Morgan fingerprints and atom counts b) Morgan fingerprints only.

Feature	Number of heavy atoms	Number of N atoms	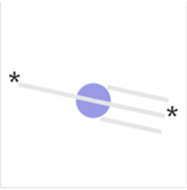	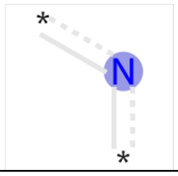	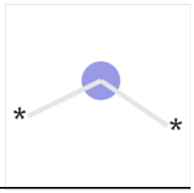
Importance	0.44	0.10	0.07	0.06	0.04

Figure S6. Top 5 most important features discriminating between QM9 and generated molecules using the optimal decision tree model. Purple circles mark the central atom in the Morgan fingerprint bits.

Additional data for model trained on OE62 training data

Generated Molecule Analysis

The OE62 training database contains 61489 molecules, out of which 53061 can be converted into valid RDKit molecules.

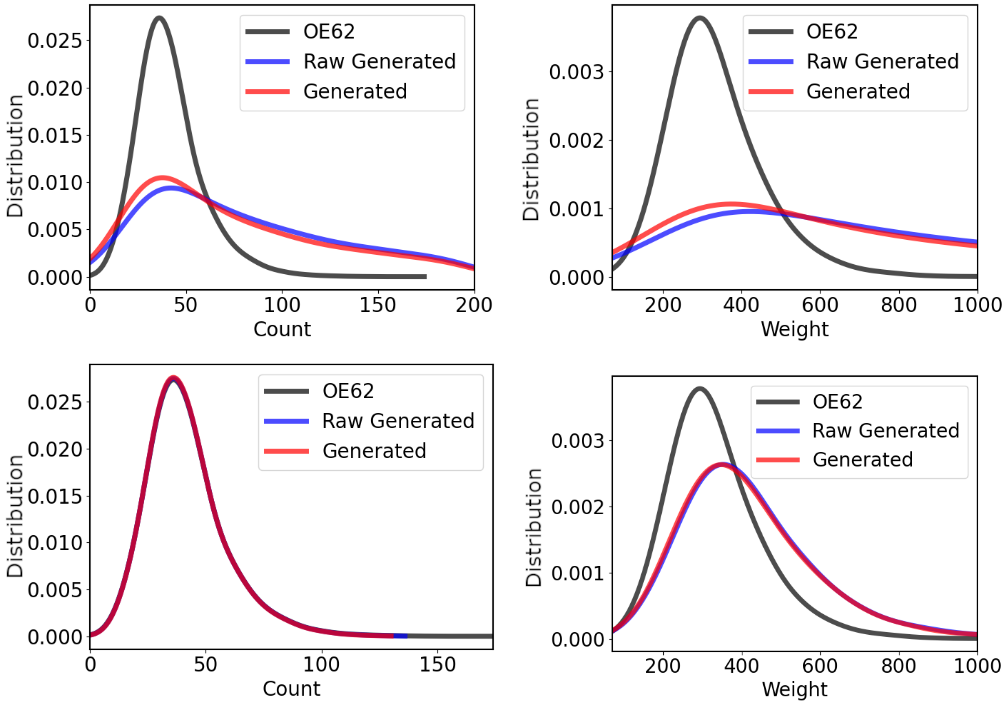

Figure S7. Distribution of total atom counts and molecular weights in OE62 training and generated (max. 200 atoms) molecules. Top panels: comparison of OE62 training data and original generated molecule database. The raw generated dataset refers to generated molecules without prior filtering of molecules according to connectivity and validity. Bottom panels: comparison of OE62 training data and the generated dataset after resampling according to the training set's atom count distribution.

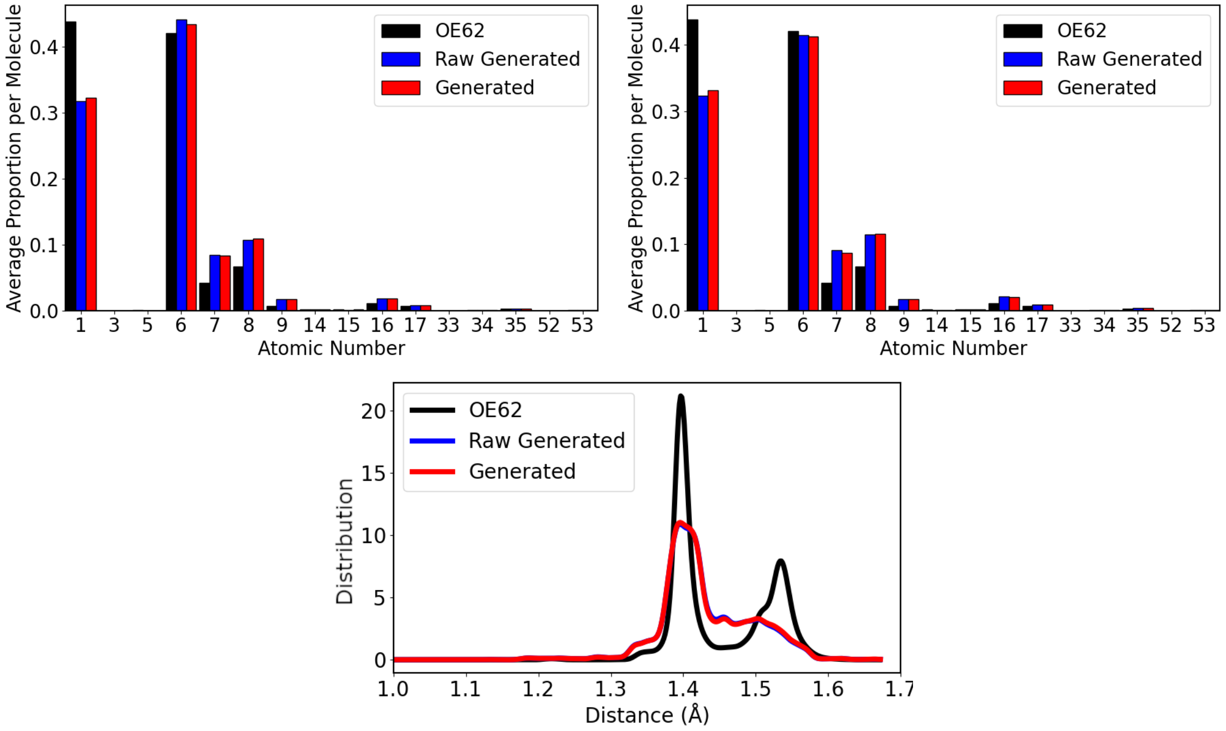

Figure S8. Top panels: Elemental composition of training data, raw generated and filtered generated structures. On the left, all generated molecules are used. On the right, the generated molecules were resampled according to the training set's atom count distribution. Bottom panel: Distribution of bonded C-C distances for the OE62 training set, the raw generated molecules, and the filtered generated molecules (both resampled).

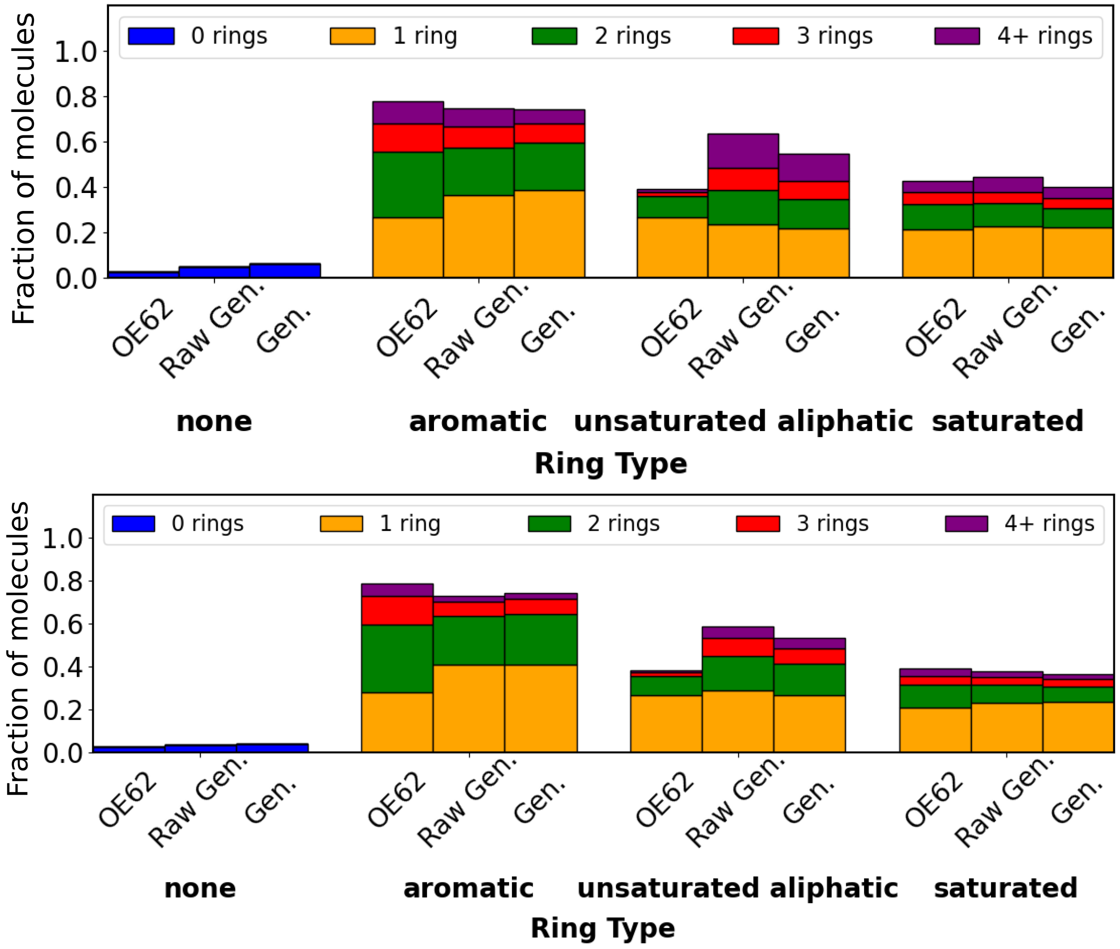

Figure S9. Proportion of molecules having different types and counts of rings in the OE62, raw generated and filtered generated databases. Top: all generated molecules, Bottom: the generated databases were resampled according to the training set's atom count distribution.

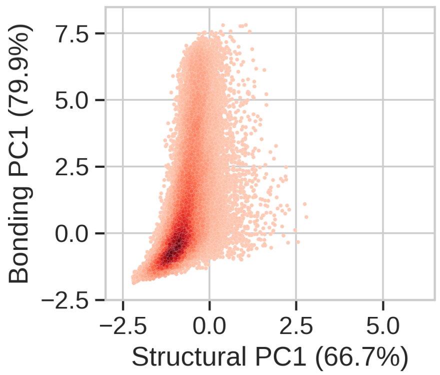

Figure S10. Latent chemical space covered by molecules generated by G-SchNet trained on OE62, filtered for validity but without any sampling with respect to the size distribution of the training database.

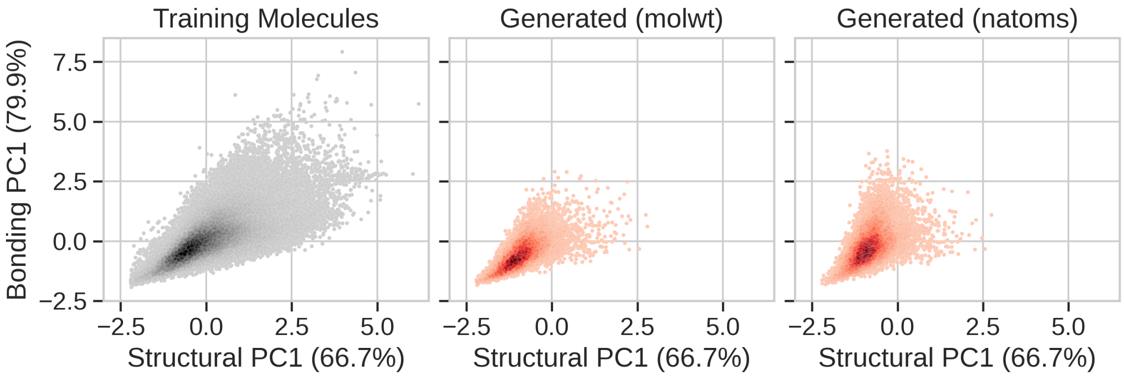

Figure S11. Latent chemical space covered by generated molecules after sampling with respect to molecular weight (molwt) and number of atoms (natoms) distributions in the OE62 training database.

Improving G-SchNet

Table S4. Number of unique and valid molecules generated with different G-SchNet settings, based on the OE62 training dataset.

	Target	Generated	Duplicate	Disconnected	Filtered	Valid RDKit
Original	60000	48051	0	1556	25945	11233
20 Å cutoffs	60000	39999	0	4750	19727	16578
10 trajectories	60000	20000	0	754	10529	8782
1:3 loss ratio	60000	19997	0	565	10625	8946

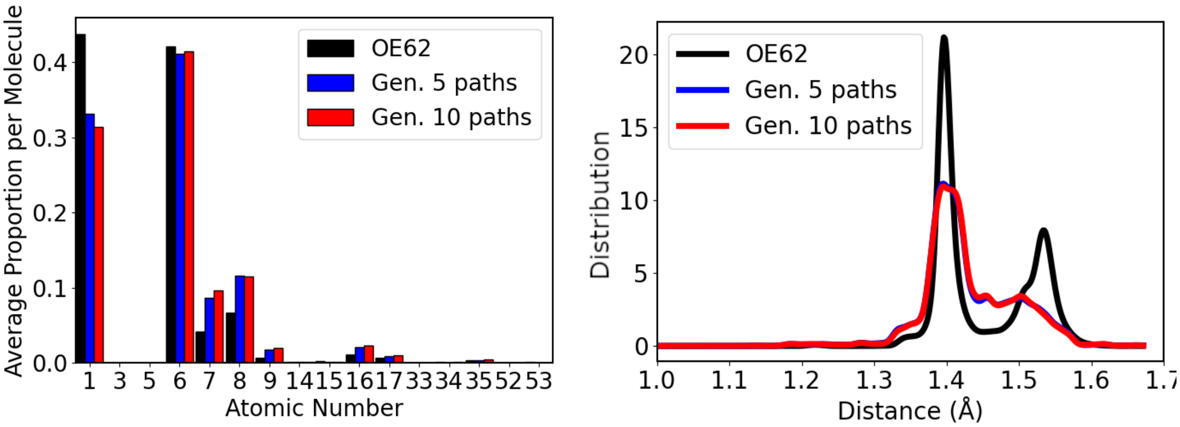

Figure S12. Elemental composition (left) and distribution of bonded C-C distances (right) for the OE62 training set and filtered generated molecules, with 5 or 10 random trajectories used for training G-SchNet, respectively. The generated databases were sampled according to the training set's atom count distribution.

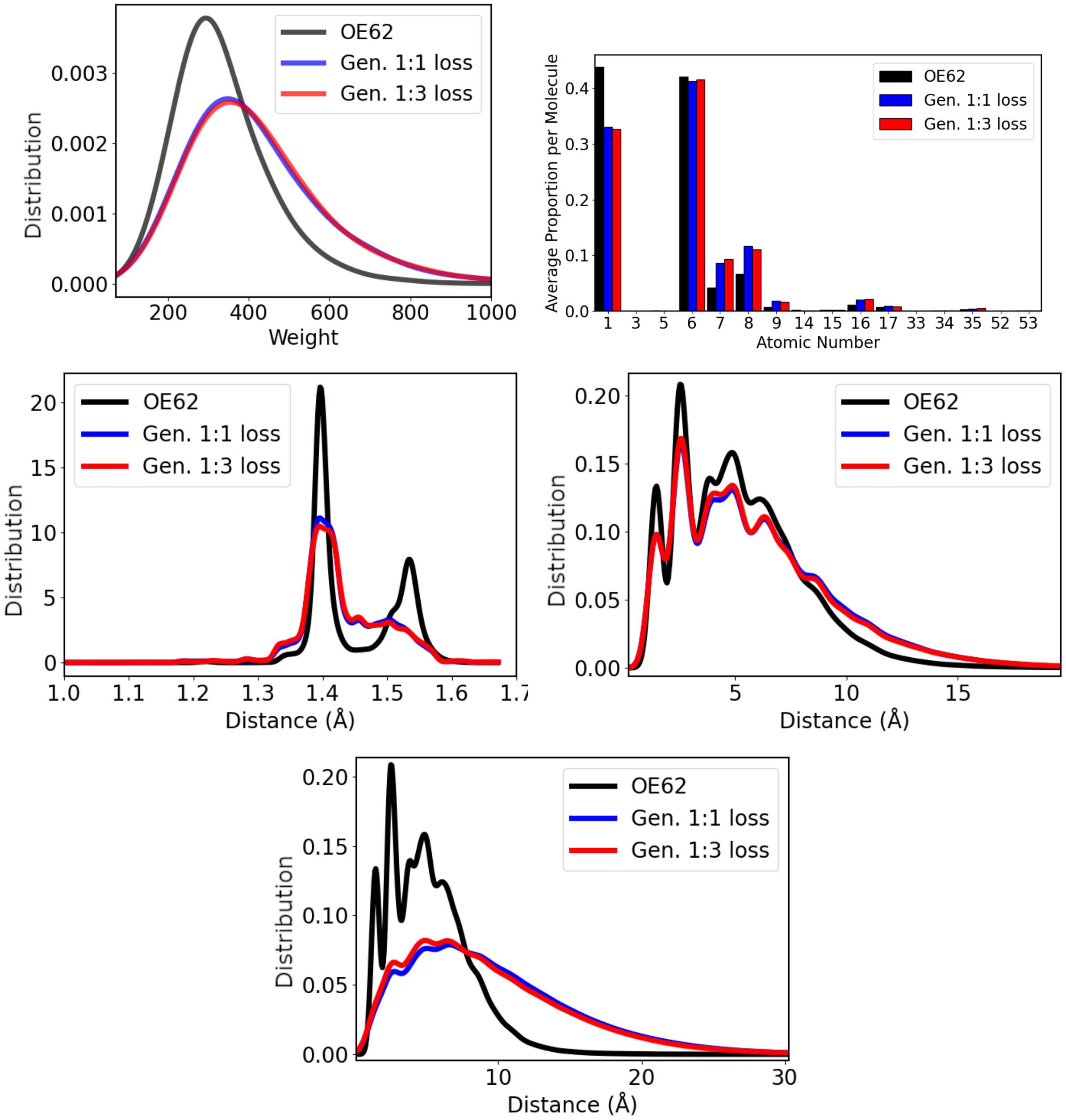

Figure S13. a) b) Elemental composition and c) distribution of bonded and d) all C-C distances for the OE62 training set and filtered generated molecules, with distance loss: element type loss ratios 1:1 or 1:3 used for training G-SchNet, respectively. The generated databases were sampled according to the training set's atom count distribution. e) all C-C distances, all generated molecules (no sampling).

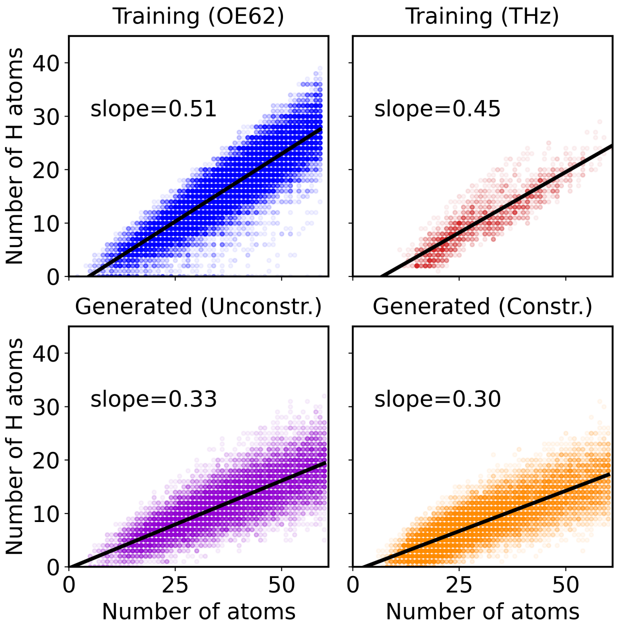

Figure S14. The effect of using a custom transform to enforce the thiolate group on the number of H atoms as a function of total number of atoms per molecule.

Decision Trees

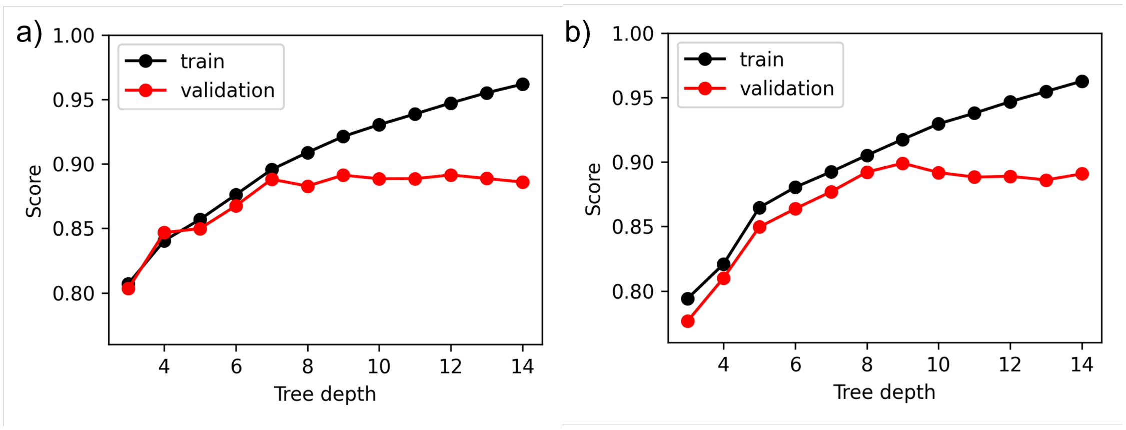

Figure S15. Optimising the tree depth parameter of a decision tree model to discriminate between OE62 and generated molecules. Features include a) Morgan fingerprints and atom counts b) Morgan fingerprints only.

When using Morgan fingerprints with radius 0 and number of bits 1000 combined with atom counts, the optimal tree depth is 13 with validation score 0.887. Without atom counts, the optimal tree depth is 9 with validation score 0.891. When using Morgan fingerprints with radius 1 and number of bits 2000, the best validation score is 0.891 for tree depth 14. Thus including atom counts in the feature vector or increasing the radius of Morgan fingerprints does not affect the score notably, and the simpler model without atom counts, fingerprints with radius 0 and number of bits 1000, and tree depth of 9 is used in further analysis. The five most important features are shown in S16.

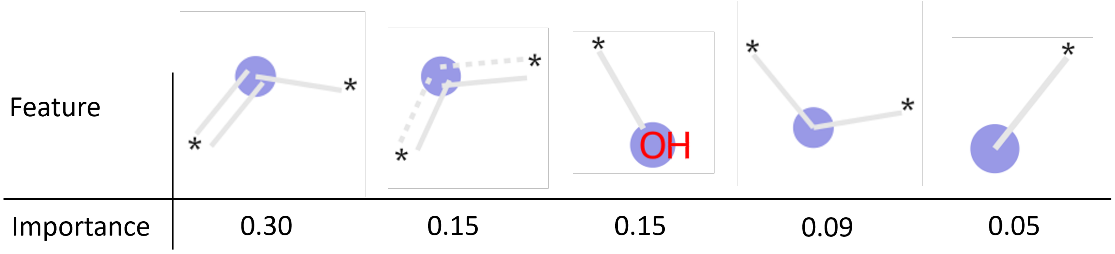

Figure S16. Top 5 most important features discriminating between OE62 and generated molecules using the optimal decision tree model. Purple circles mark the central atom in the Morgan fingerprint bits.
